# Recurrent Rhabdomyolysis Induced by a Viral Illness in a Young Patient

**DOI:** 10.7759/cureus.52625

**Published:** 2024-01-20

**Authors:** Zahid Khan, Osman Ahmed, Syed Aun Muhammad, Jonard Carpio

**Affiliations:** 1 Acute Medicine, Mid and South Essex NHS Foundation Trust, Southend-on-Sea, GBR; 2 Cardiology, Barts Heart Centre, London, GBR; 3 Cardiology and General Medicine, Barking, Havering and Redbridge University Hospitals NHS Trust, London, GBR; 4 Cardiology, Royal Free Hospital, London, GBR; 5 Respiratory Medicine, Mid and South Essex NHS Foundation Trust, Southend-on-Sea, GBR; 6 Cardiology, Mid and South Essex NHS Foundation Trust, Southend-on-Sea, GBR; 7 Internal Medicine, Mid and South Essex NHS Foundation Trust, Southend-on-Sea, GBR

**Keywords:** acute rhabdomyolysis, creatinine kinase-muscle/brain, rhabdomyolysis, infectious rhabdomyolysis, exertional rhabdomyolysis

## Abstract

Rhabdomyolysis is a syndrome caused by skeletal muscle disruption that results in the release of muscle proteins into circulation, which can lead to life-threatening systemic complications. These complications include acute kidney injury (AKI), renal failure, compartment syndrome, and disseminated intravascular coagulopathy. Patients commonly present with muscle pain, fatigue, weakness, and dark-colored urine. We present the case of a 37-year-old male who presented to the hospital with pain in the lower limbs and difficulty in mobility for the past two days after returning from Jamaica. He had a mild cold and body aches but denied any sore throat, cough, or shortness of breath (SOB). He tested negative for COVID-19. He had attended his local hospital the previous night, but due to the long waiting time, he presented to the accident and emergency department at our hospital. His physical examination was normal, and his urine was dark in color. All laboratory test results were normal, except for creatinine kinase (CK) levels >100,000 IU/L (reference: 40-320 IU/L) and an alanine transaminase (ALT) level of 376 U/L (reference: 30-130 U/L). Magnetic resonance imaging of both femurs revealed a high signal in multiple muscle compartments bilaterally on a short TI inversion recovery (STIR) sequence. Autoimmune screening results were negative. He had a similar episode last year due to COVID-19 with elevated CK levels. He received conservative treatment with IV fluids and was discharged eight days after hospital admission.

## Introduction

Rhabdomyolysis is caused by muscle breakdown, resulting in the release of muscle proteins into the blood, often secondary to a precipitating factor [1]. Most cases of rhabdomyolysis are caused by viral or bacterial infections, although other precipitating factors have also been reported [1,2]. Creatine kinase (CK) is the most sensitive and useful indicator of muscle injury in patients with rhabdomyolysis [3]. Patients with suspected COVID-19 have been reported to present with recurrent rhabdomyolysis [1,4]. Viral infections are a common trigger for infection-induced rhabdomyolysis, and some patients experience recurrent rhabdomyolysis due to viral infections [1]. Exercise is another common trigger for this condition, and severe rhabdomyolysis can cause life-threatening conditions including renal failure, hyperkalemia, compartment syndrome, and disseminated intravascular coagulopathy. Myocyte injury is followed by the efflux of intracellular contents into circulation, which can result in hyperkalemia, hyperuricemia, and hyperphosphatemia [3]. Severe rhabdomyolysis can result in lactic acid-induced metabolic acidosis, secondary to muscle ischemia [1,3].

As striated muscles become damaged, plasma CK is released into circulation, and its concentration is proportional to the extent of striated muscle injury. A CK concentration of at least five times the upper limit of normal is expected in patients with rhabdomyolysis, and a CK level > 50,000 U/L is associated with an approximately 50% incidence of acute renal failure [1,3]. Urinalysis is not reliable in patients with rhabdomyolysis, as routine dipstick detects heme; hence, it may not be able to differentiate between hematuria, hemoglobinuria, and myoglobinuria [1,2]. Muscle disintegration releases myoglobin into circulation, causing myoglobinuria, which results in brownish discoloration of the urine. We present the case of a 37-year-old patient who presented with severe lower-limb weakness, fatigue, and dark-colored urine secondary to rhabdomyolysis.

## Case presentation

A 37-year-old patient presented to the hospital with mainly lower limb weakness, fatigue, and difficulty walking for the past few days. His past medical history was significant for COVID-19-induced rhabdomyolysis a year prior to the current presentation. His current symptoms started a day after returning from a two-week holiday in Jamaica. He had mild cold-like symptoms and body aches but denied any chest pain, shortness of breath (SOB), cough, or fever. He underwent two COVID-19 tests at home, both yielding negative results. His vital signs were as follows: blood pressure (BP) 130/65 mmHg, heart rate (HR) 89 bpm, respiratory rate (RR) 19, and oxygen saturation (SpO2) 98% on room air. Physical examination results were normal, except for proximal weakness in the lower limbs, with power in the lower limbs being 4/5.

Laboratory tests showed an elevated CK level of >100,000 U/L (Ref: <320 U/L), C-reactive protein (CRP) 28 (Ref: <5 mg/L), and alanine transaminase (ALT) 376 (Ref: < 50 U/L) (Table 1). His CK, ALT, and CRP levels during his previous admission were 98,000 U/L, 476 U/L, and 5 mg/L, respectively. He was conservatively managed with intravenous fluids, ibuprofen 400 mg three times daily, and co-codamol 30/500 mg four times daily. His CK level improved after a week of treatment. Autoimmunity tests were negative except for weakly positive antinuclear antibody (HEp2) 1:80, Complement C4 0.90 (Ref: 0.14 - 0.54 g/L), and gamma-glutamyl transferase 82 (Ref: < 55 U/L), as shown in Table 2. Urinalysis demonstrated a urine albumin-to-creatinine ratio of <1 and a total protein-to-creatinine ratio of 29.8 (Ref: < 15 mg/mmol). Tests for anti-Jo antibodies, anti-Mi-2, anti-MDA-5, anti-TIF-1, anti-NXP-2, and anti-SAE antibodies for polymyositis and dermatomyositis were negative.

**Table 1:** Patient lab results during hospital admission.

Blood test	Day 1	Day 2	Day 4	Day 6	Day 8	Normal range
Hemoglobin	155	140	135	132	141	133-173 g/L
White cell count	5.2	4.5	4.5	4.2	4.1	3.8-11 × 10^9^/L
Neutrophil count	2.78	2.38	2.63	2.03	1.88	1.7-7.5 × 10^9^/L
Platelet count	321	299	292	271	262	150-400 × 10^9^/L
C-reactive protein	28	18	16	15	08	0-5 mg/L
Creatinine Kinase	>100,000	71153	55507	29776	11206	<320 U/L
Sodium	137	139	138	139	140	133-146 mmol/L
Potassium	4.9	4.5	4.4	4.2	4.3	3.5-5.3 mmol/L
Urea	6.0	5.4	4.6	3.7	4.3	2.5-7.8 mmol/L
Creatinine	80	67	62	67	65	59-104 umol/L
Albumin	43	38	-	-	38	35-50 g/L
Alanine transaminase	376	290	-	-	208	<50 U/L
Alkaline phosphatase	85	78	-	-	72	30-130 U/L
Total protein	74	70	-	-	64	60-80 g/L
Vitamin B12	-	-	-	225	-	120-900 ng/L
Transferrin saturation %	-	-	-	50	-	15-50%
Iron	-	-	-	25.4	-	10-30 µmol
Transferrin	-	-	-	2.04	-	2.0-3.6 g/L
Thyroid stimulating hormone	-	-	-	0.84	-	0.3-5.0 mU/L
Free T4	-	-	-	12.3	-	7.9-16 pmol/L

**Table 2:** Autoimmune screening results for the patient.

Lab test	Value	Reference range
Cyclic citrullinated peptide (CCP) IgG antibodies	0.9	0.4-6.9 U/mL
Antinuclear antibody (Hep2)	1:80 (weakly positive)	-
Anti-smooth muscle antibody	Negative	<1:40
Anti-mitochondrial antibody	Negative	<1:40
Anti-LKM (liver-kidney microsomal) antibody	Negative	<1:40
Rheumatoid factor	<10	0-14 U/mL
Lactate dehydrogenase (LDH)	1670	240-480
Aspartate transaminase (AST)	376	<50 U/L
Complement C3	1.5	0.90-1.80 g/L
Complement C4	0.9	0.14-0.54 g/L
Erythrocyte sedimentation rate (ESR)	21	2-28 mm/hour
Gamma-glutamyl transferase	82	<55 U/L
Antineutrophil Cytoplasmic Antibodies (ANCA)	Negative	<1:20
Anti-double-stranded deoxyribonucleic acid antibodies (Anti-ds DNA)	1.2	0.5-9.9 IU/mL
HIV antigen/antibody	Not detected	No evidence of infection if not detected
Hepatitis C antigen/antibody	Not detected	No evidence of infection if not detected
Hepatitis B antigen/antibody	Not detected	No evidence of infection if not detected

**Figure 1:**
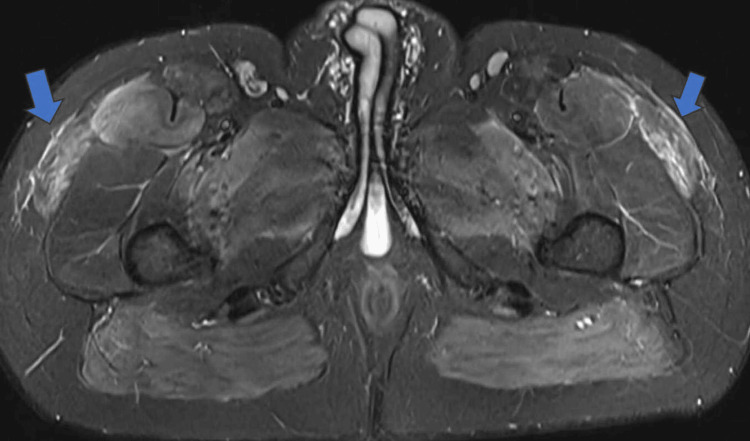
MRI scan of the thighs showing high signal intensity (blue arrows).

**Figure 2:**
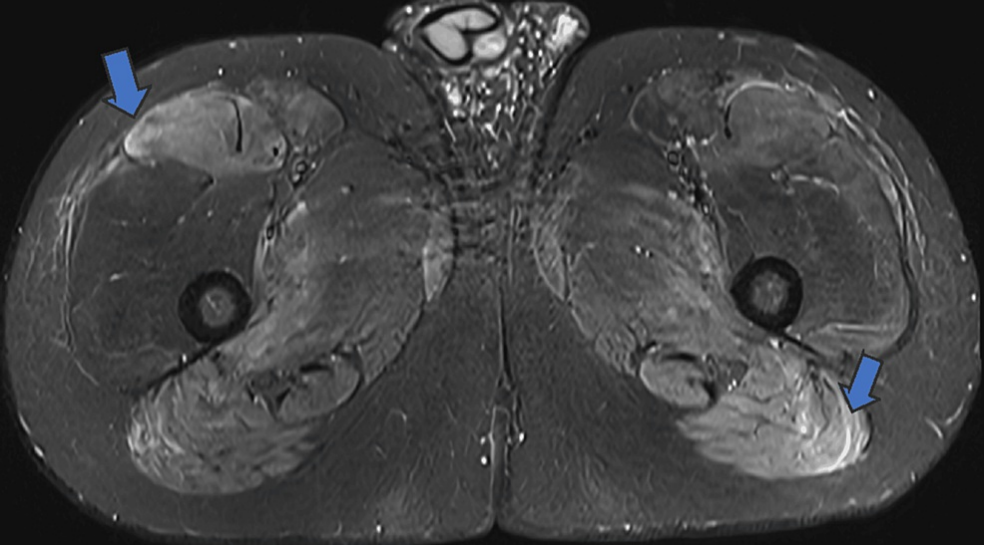
MRI shows bilateral high signal intensity, suggestive of acute myositis (blue arrows).

**Figure 3:**
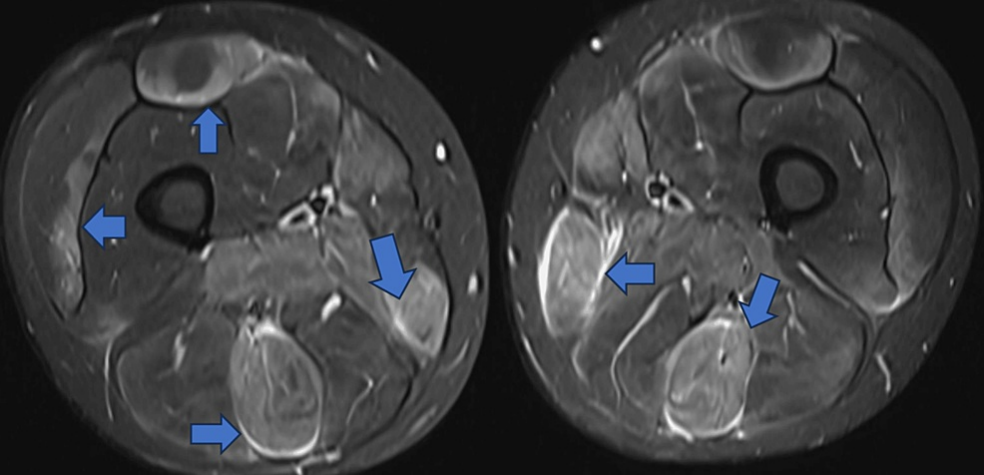
MRI shows areas of high signal intensity, suggestive of acute myositis, indicated by blue arrows.

MRI of the femurs demonstrated a high signal in multiple muscle compartments bilaterally on the short-TI inversion recovery (STIR) sequence. The affected muscles included the adductors, hamstrings, gracilis, sartorius, and tensor fascia latae, bilaterally (Figures 1-3). Ultrasound of the abdomen showed normal liver texture and normal-shaped kidneys.

He stayed in the hospital for a week, the CK level dropped to below 11000 after a week of treatment with intravenous fluids, and was discharged home with outpatient follow-up. He was followed up in the outpatient clinic and has remained well since then.

## Discussion

Rhabdomyolysis is a common complication of infections, drugs, intense exercise, and heat stress [4,5]. Its presentation varies from asymptomatic to life-threatening; the main abnormalities detected include electrolyte derangement, impairment of renal function, and, rarely, disseminated intravascular coagulation (DIC) [1,6]. Rhabdomyolysis can be caused by trauma or direct injury, drugs, hyperthermia, infections, toxins, muscle ischemia, prolonged bed rest, or metabolic or genetic conditions [6]. Viral infections are believed to induce rhabdomyolysis due to the invasion of muscle tissue, causing toxin-induced muscle damage, an innate inflammatory response, or a combination of these mechanisms. Additionally, hyperthermic states such as malignant hyperthermia and neuroleptic malignant syndrome can also cause rhabdomyolysis [5]. The most common denominators of both traumatic and non-traumatic rhabdomyolysis are myalgia, swelling, limb weakness, dark-colored urine, or gross pigmenturia without hematuria, which occur as a result of muscle necrosis [5]. Patients with virus-induced rhabdomyolysis have elevated CK levels in blood tests [7]. Clinically, rhabdomyolysis is characterized by a triad of symptoms: weakness, myalgia, and myoglobinuria. However, this triad is observed in less than 10% of patients, and over 50% of patients present with discolored urine as the initial symptom without any obvious muscle pain or weakness [4,5].

The most sensitive marker for identifying muscle injury that can lead to rhabdomyolysis is an elevated CK level in the absence of cardiac and brain injury. The exact level of CK associated with the severity of muscle damage and renal failure has shown mixed results; however, a level > 5,000 IU/L is likely to result in muscle damage, and these patients should receive vigorous fluid hydration, stop any offending drugs, and treat the precipitating factor [5,8,9]. Rhabdomyolysis has been associated with COVID-19 and other viral illnesses in several case reports [10-13]. There appears to be no direct association between the severity of COVID-19 and the risk of rhabdomyolysis. Patients with COVID-19 who required intensive care and prolonged hospital admissions were more likely to develop rhabdomyolysis, possibly because these patients were critically ill. Similarly, patients with rhabdomyolysis and COVID-19 are more likely to require renal replacement therapy, and the prognosis is more favorable in patients with COVID-19, rhabdomyolysis, and normal renal function [1,14,15].

A study based on 38 COVID-19 case reports found that 34 of the 38 patients were male, and 7 of the 17 cases of Afro-Caribbean ethnicity presented with an average CK level of 70,250 IU/L. Two patients required hemodialysis before discharge, and 17 died. It is not entirely clear why some patients are at a higher risk of developing rhabdomyolysis following COVID-19 infection than others; however, older patients are at a higher risk. There has been no correlation with ethnicity in published case reports [1]. Recurrent rhabdomyolysis has been observed in a few young Afro-Caribbean patients due to viral illnesses, with CK levels exceeding 100,000 IU/L, and no other cause has been identified in these patients despite thorough workup [1,13]. Our patient also underwent an extensive workup, but no other cause of recurrent rhabdomyolysis could be identified apart from the viral illness. Our patient had normal renal function and completely recovered from the illness with conservative treatment.

## Conclusions

Rhabdomyolysis induced by viral infections is a rare condition with a good prognosis when treated promptly. Patients with rhabdomyolysis who have normal renal function generally tend to have favorable outcomes. It is advisable to screen patients with recurrent rhabdomyolysis for autoimmune conditions to rule out other causes of the recurrence. In cases of drug-induced rhabdomyolysis, discontinuing the offending drug and providing vigorous fluid therapy and rehydration are essential.
